# List of Questions to Be Answered by the Governors of Hospitals, &c.

**Published:** 1834-04-01

**Authors:** 


### INTELLIGENCE.

A List of Questions has been circulated, which the governors of hospitals, dispensaries, and infirmaries, are required to answer for the information of the Committee appointed by the House of Commons to inquire into the state of MEDICAL PRACTICE AND EDUCATION.

